# Receptor activity–modifying protein 3 enhances GLP-1-mediated insulin secretion

**DOI:** 10.1016/j.jbc.2025.110604

**Published:** 2025-08-18

**Authors:** Abigail Pearce, Poonam Kumari, Claudia M. Sisk, Matthew Harris, Ho Yan Yeung, Sabrina Winfield, Kathleen M. Caron, Graham Ladds

**Affiliations:** 1 Department of Pharmacology, University of Cambridge, Cambridge, UK; 2 Department of Cell Biology and Physiology, School of Medicine, University of North Carolina at Chapel Hill, Chapel Hill, North Carolina, USA

**Keywords:** G protein–coupled receptor, allosteric regulation, diabetes, second messenger, insulin secretion, calcium intracellular release

## Abstract

The targeting of the glucagon-like peptide-1 (GLP-1) receptor for diabetes and obesity is not a novel strategy, with recent therapeutics showing efficacy in weight loss and glycemic control. However, they are also associated with side effects, including gastrointestinal disruptions and pancreatitis. Developing agonists with different signaling profiles or that exert some tissue selectivity can circumvent these on-target, unwanted effects. Receptor activity–modifying proteins (RAMPs) offer the potential to do both, through modulation of agonist binding and signaling, as well as surface expression. The GLP-1 receptor was found to interact with RAMP3, with the heterodimer able to bind agonists at the cell surface. RAMP3 expression biased the receptor toward Ca^2+^ mobilization, away from the canonical cAMP-driven signaling. When examining G protein coupling, the interaction with RAMP3 reduced activation of the cognate Gα_s_ but increased secondary couplings to Gα_q_ and Gα_i_. These increased couplings led to an elevation in glucose-stimulated insulin secretion when cells overexpressing RAMP3 were stimulated with GLP-1. A reciprocal effect was observed when looking at reduced expression of endogenous RAMP3, with a loss of sensitivity to GLP-1 in both glucose and insulin tolerance tests in a *Ramp3* KO mouse model. The effects of this interaction can then inform the selection of models and peptide design when targeting this receptor for therapeutic intervention.

Obesity and type 2 diabetes mellitus (T2DM) are increasingly prevalent diseases, with the incidence expected to rise to 51% of the global population by 2035 (https://data.worldobesity.org/publications/?cat=19). Finding treatments for these diseases has been an ongoing mission, with efforts more recently focused on simultaneous targeting of both conditions, with the aim to improve T2DM by resolving the underlying obesity.

A recent therapeutic advance has been the approval of peptides targeting the glucagon-like peptide-1 (GLP-1) receptor (GLP-1R) for obesity and weight loss. The incretin effect, whereby oral glucose elevates plasma insulin levels to a greater degree than intravenous administration, was first observed in 1964 (1). This is mediated by incretin hormones, the best known of which are GLP-1 and the glucose-dependent insulinotropic polypeptide (GIP). As such, mimetics of the endogenous GLP-1 hormone, the first of which was exenatide (exendin-4), were approved for T2DM in 2005 (2). However, newer mimetics semaglutide (trade names of Ozempic and Wegovy) or tirzepatide (sold under Mounjaro or Zepbound), the latter of which targets both the GLP-1R and the GIP receptor (GIPR), have garnered much attention through their dual effects on lowering plasma glucose and aiding weight loss. Semaglutide has since been reported to protect against neurodegeneration and neuroinflammation (3) as well as cardiovascular disease (4). This culminated in semaglutide being labeled as the 2023 breakthrough of the year (5). However, with their increased use, attention is being drawn to the unwanted side effects of GLP-1 mimetics. Common side effects are nausea and vomiting as well as other gastrointestinal discomforts. However, more serious side effects are also known, such as pancreatitis, kidney failure, and gallbladder problems (6). There is therefore the unmet need to develop mimetics with improved therapeutic potential that exhibit fewer side effects.

One method of reducing on-target unwanted effects is to selectively activate signaling pathways associated with the positive outcome, relying on the concept of signaling bias. The GLP-1R is a class B1 G protein–coupled receptor (GPCR), which predominantly couples to Gαs, leading to an increase in cAMP production. However, like other members of class B1, the GLP-1R exhibits pleiotropy, coupling to other Gα families and recruiting β-arrestins (7, 8), leading to a multifaceted signaling output. For other pleiotropic GPCRs, the activation of these different pathways can be influenced by the agonist, giving rise to signaling bias. This has been studied most heavily for the μ-opioid receptor, where it is thought that agonist tolerance is mediated by β-arrestins, leading G protein–biased agonists to be preferred (9).

Signaling bias can also be influenced by the tissue because of differential expression of signaling proteins as well as allosteric modulators. A common protein allosteric modulator of class B1 GPCRs is the receptor activity–modifying proteins (RAMPs). First discovered for the calcitonin-like receptor (CLR), with which they form obligate heterodimers, RAMPs influence the expression, affinity, and signaling bias of the receptor. For the calcitonin family, this leads to receptors considered functionally distinct (10, 11), although smaller effects are observed on other receptors, for example, the vasoactive intestinal peptide receptor 1 or glucagon receptor (GCGR) when coexpressed with RAMP2 (12). Importantly, the effects of RAMPs are agonist and pathway dependent, with RAMP2 increasing Gα_s_ coupling at GCGR in response to glucagon and oxyntomodulin (an endogenous dual agonist of both GCGR and GLP-1R) but decreasing the response to GLP-1 and liraglutide (a synthetic, lipidated GLP-1 mimetic, approved in the treatment of T2DM) (13).

RAMPs have been implicated in diabetes and obesity, although their role appears multifaceted. Of the GPCRs that interact with RAMPs, many are involved in the regulation of glucose levels and body weight. The pancreatic hormone amylin binds a class B1 GPCR, with the three amylin receptor heterodimers between the calcitonin receptor and RAMP1–3. RAMPs are expressed in many of the tissues of the endocrine system, such as the thyroid and hypothalamus, and global RAMP KO in rodents causes dysregulation of body weight and glycemic control (14, 15). (The role of RAMPs in diabetes and obesity is further reviewed in the study by Malcharek *et al.* (16)). Importantly, RAMPs are coexpressed with the incretin hormone receptors across regions of the brain and the pancreas (Human Protein Atlas as of January 2025). The interaction between GIPR and RAMPs and the consequences for glucose tolerance have been studied previously (17), but the involvement of GLP-1R is less known. GLP-1R has been previously shown to interact with all three RAMPs (18, 19), with depressive effects on cAMP accumulation observed. However, specific interactions at the plasma membrane have neither been studied nor have any effects of RAMP expression on physiological roles of the receptor, such as glucose-stimulated insulin secretion (GSIS).

Herein, we measured a specific membrane interaction between GLP-1R and RAMP3 but not RAMP1 or 2. The two proteins were observed to form a complex able to bind agonists, but no effect was observed on the affinity of the receptor. RAMP3 expression decreased cAMP accumulation and increased mobilization of Ca^2+^ from intracellular stores (Ca^2+^)_i_. This was determined to be primarily because of an increase in Gα_i/o_ coupling. In contrast, no effect was observed on internalization, with only a decrease in maximal β-arrestin recruitment. This elevation in (Ca^2+^)_i_ was shown to lead to enhanced GSIS. The reciprocal effect was observed in murine cell and animal models, where a knockdown or a KO of *Ramp3* decreased insulin secretion in response to GLP-1. There is therefore the potential for improved GLP-1 mimetics, which show bias toward the GLP-1R–RAMP3 complex, with subsequent effects on tissue selectivity and therapeutic profile.

## Results

### GLP-1R specifically interacts with RAMP3

**Figure 1:**
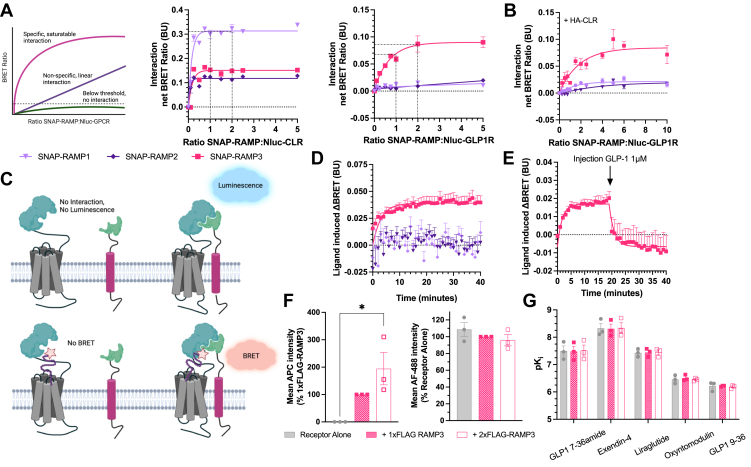
**GLP-1R and RAMP3 interact and can bind agonists as a complex.***A*, cell-surface NanoBRET assay measuring interactions between Nluc–CLR (*left*) and Nluc–GLP-1R (*right*) with SNAP-RAMPs labeled with SNAP-Surface Alexa Fluor 488 in Cos7 cells, n = 4. *B*, Nluc–GLP-1R interaction with labeled SNAP-RAMPs, in the presence of a fixed concentration of HA-CLR, n = 8. *C*, schematic of a NanoBiT assay for measuring GLP-1R–RAMP interactions and fluorescent ligand binding to the complex. *D*, binding of Ex-4-Red to the LgBiTGLP-1R SmBiT–RAMP3 complex in HEK293T cells (n > 4), which can then be displaced by addition of 1 μM unlabeled GLP-1 *E*, n = 3. *F*, expression of FLAG-RAMP3 (*left*) or Nluc–GLP-1R (*right*) in HEK293T cells transfected with Nluc–GLP-1R and increasing amounts of FLAG-RAMP, n = 3. *G*, binding affinity (p*K*_i_) values for GLP-1, exendin-4, liraglutide, oxyntomodulin, and GLP-1 (9–36) at Nluc–GLP-1, coexpressed with FLAG-RAMP3, n = 3. Mean values of n experimental replicates are shown as ±SEM, and statistical differences were determined through Kruskal–Wallis test for nonparametric data or one-way ANOVA, *∗p* < 0.05. BRET, bioluminescence resonance energy transfer; CLR, calcitonin receptor–like receptor; GLP-1, glucagon-like peptide-1; GLP-1R, GLP-1 receptor; HEK293T, human embryonic kidney 293T cell line; RAMP, receptor activity–modifying protein.

Although GLP-1R expression has been unable to promote RAMP membrane trafficking, interactions have been observed at the molecular level, for example, using bioluminescence resonance energy transfer (BRET) (19). Therefore, to determine if these observed interactions are present at the plasma membrane, a cell surface BRET interaction assay was used as previously described (17). Nluc-GPCR and SNAP-RAMP were transiently transfected into Cos7 cells, which do not endogenously express RAMPs, calcitonin receptor, or CLR (20), to reduce any interference from unlabeled proteins. SNAP-RAMPs were irreversibly conjugated to SNAP-Surface Alexa Fluor 488 to specifically measure cell surface interactions; the conjugate was cell impermeable, so only labeled SNAP-RAMPs were expressed at the cell surface. All three RAMPs displayed an interaction with CLR, as observed by the saturating increase in BRET ratio. GLP-1R only displayed a saturating increase in BRET with SNAP-RAMP3 (Fig. 1*A*). Overexpression of HA-CLR was not shown to promote an interaction between Nluc–GLP-1R and SNAP-RAMP1 or 2, indicating that the expression of RAMP at the membrane is not sufficient to induce an interaction, even when increasing the amount of SNAP-RAMP 10-fold over Nluc–GLP-1R.

To validate the formation of a GLP-1R–RAMP3 complex, we measured binding of a fluorescent ligand to the protein heterodimer. We utilized NanoBiT, with LgBiT preceding the N terminus of the receptor, and SmBiT upstream of the N terminus of the RAMP (illustrated in Fig. 1*C*). Binding of the Tag-Lite GLP-1R red ligand, based on exendin-4 (Ex-4-Red), was observed through an increase in the BRET ratio, with a calculated *K*_*d*_ of 6.7 nM (Fig. 1*D*). This was not observed for SmBiT-RAMP1 or -RAMP2. The BRET ratio could be reduced by injection of 1 μM unlabeled GLP-1, competing with the fluorescent agonist and further demonstrating binding had occurred to the heterodimer (Fig. 1*E*).

### FLAG-RAMP3 has no effect on GLP-1R expression or agonist binding

When measuring their effects on GPCR signaling, RAMPs are commonly coexpressed at a 1:1 ratio because of the stoichiometry of the interaction with CLR (21). However, the maximal interaction between GLP-1R and RAMP3 in the BRET assay was observed when RAMP3 was expressed at twice that of GLP-1R (by the weight of expression plasmid, Fig. 1*A*). We therefore measured the effect of increasing RAMP3 expression on GLP-1R, either coexpressing (1:1 ratio) or overexpressing (2:1 ratio) RAMP3. It was confirmed that the expression of FLAG-RAMP3 was increased at the 2:1 ratio compared with 1:1, but no difference was observed on GLP-1R expression (Fig. 1*F*).

In addition to receptor expression, differences in signaling could be driven by changes in affinity. As RAMP modulation of GPCR signaling can be agonist dependent, effects were examined across a range of agonists. GLP-1 and oxyntomodulin are endogenous agonists, the latter with a reduced potency. GLP-1(9–36), a product of the degradation of GLP-1, which can show weak partial agonism, was also tested. Examples of incretin mimetics, exendin-4 and liraglutide, were also included. FLAG-RAMP3 had no influence on binding affinity for any peptide as measured through a NanoBRET competition binding assay (Fig. 1*G*).

### Second messenger signaling is influenced by RAMP3 expression

**Figure 2:**
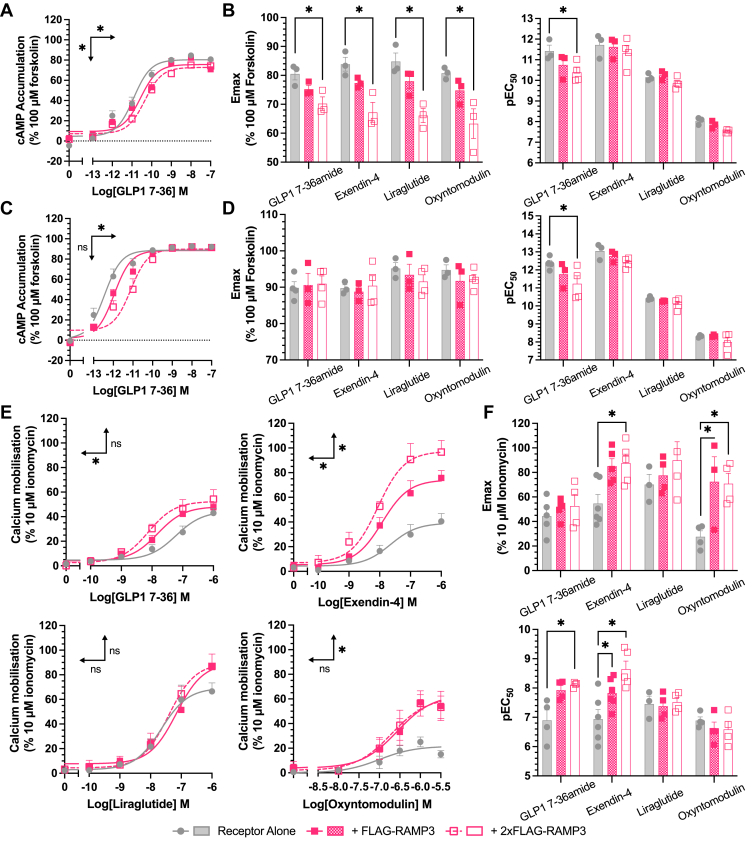
**RAMP3 decreases cAMP accumulation but increases (Ca^2+^)_i_ mobilization.** cAMP accumulation mediated by Nluc–GLP-1R, expressed with increasing concentrations of FLAG-RAMP3, in response to 8-min stimulation with GLP-1 (*A*), with *E*_max_ and pEC_50_ values plotted for GLP-1, exendin-4, liraglutide, or oxyntomodulin (*B*). *C* and *D*, as before but for 30-min stimulation. Responses are normalized to 100 μM forskolin, n > 3. *E*, mobilization of (Ca^2+^)_i_ by Nluc–GLP-1R with FLAG-RAMP3, in response to stimulation with GLP-1, exendin-4, liraglutide, or oxyntomodulin, with *E*_max_ and pEC_50_ values plotted (*F*). Responses are normalized to 10 μM ionomycin, n > 3. Mean values of n experimental replicates are shown as ±SEM; significance between RAMP conditions was determined through one-way ANOVA, ∗*p* < 0.05. *Horizontal arrows* indicate changes in potency (pEC_50_), and *vertical arrows* designate changes in efficacy (*E*_max_). (Ca^2+^)_i_, intracellular Ca^2+^ concentration; GLP-1, glucagon-like peptide-1; GLP-1R, GLP-1 receptor; RAMP, receptor activity–modifying protein.

As with most class B1 GPCRs, the primary signaling mediator for the GLP-1R is Gα_s_, leading to an increase in cAMP (7, 22). When stimulated with GLP-1 for 8 min, a dose-dependent increase in cAMP was observed (pEC_50_ value of 11.4 ± 0.3). This was reduced by RAMP3 expression, with a reduction in the potency at 8 min when RAMP3 was overexpressed (to 10.4 ± 0.2) (Fig. 2*A*, Table 1). The pattern was preserved across exendin-4, liraglutide, and oxyntomodulin, with reductions in both potency and efficacy observed when RAMP3 was overexpressed (Fig. 2*B*). GLP-1(9–36) did not elicit sufficient cAMP production over the concentration range tested to calculate potency and efficacy values.

**Table 1:** Differential effects of RAMP3 expression on second messenger signaling of the GLP-1R

	cAMP 8 min				cAMP 30 min				Ca^2+^ mobilization			
	pEC_50_	*E*_max_	LogRA	n	pEC_50_	*E*_max_	LogRA	n	pEC_50_	*E*_max_	LogRA	n
GLP-1 (7–36 amide)												
Receptor alone	11.4 ± 0.3	80.5 ± 2.1	0.00 ± 0.16	3	12.4 ± 0.2	88.8 ± 1.7	0.00 ± 0.16	4	6.9 ± 0.4	44.4 ± 6.4	0.00 ± 0.27	6
+ FLAG-RAMP3	10.8 ± 0.4	75.2 ± 1.3	−0.37 ± 0.20	3	11.8 ± 0.4	90.6 ± 1.4	−0.49 ± 0.14	3	7.9 ± 0.2	50.6 ± 3.5	0.64 ± 0.25	5
+ 2xFLAG-RAMP3	10.4 ± 0.2∗	70.19 ± 2.3∗	−0.61 ± 0.16 ∗	3	11.2 ± 0.4∗	91.0 ± 2.1	−1.34 ± 0.17 ∗	4	8.1 ± 0.0∗	52.6 ± 9.3	0.97 ± 0.27∗	5
Exendin-4												
Receptor alone	11.7 ± 0.3	83.9 ± 2.4	0.00 ± 0.16	3	13.1 ± 0.3	89.7 ± 0.9	0.00 ± 0.17	3	6.9 ± 0.3	54.7 ± 7.3	0.00 ± 0.39	6
+ FLAG-RAMP3	11.6 ± 0.3	77.4 ± 1.0	−0.21 ± 0.16	3	12.8 ± 0.2	88.8 ± 1.4	−0.33 ± 0.17	3	7.8 ± 0.2∗	85.1 ± 6.0	0.52 ± 0.33	6
+ 2xFLAG-RAMP3	11.3 ± 0.3	67.2 ± 3.5∗	−0.61 ± 0.16 ∗	3	12.4 ± 0.1	90.4 ± 2.8	−0.50 ± 0.14	4	8.6 ± 0.3∗	87.6 ± 7.2∗	0.76 ± 0.33	6
Liraglutide												
Receptor alone	10.2 ± 0.1	84.8 ± 2.9	0.00 ± 0.13	3	10.4 ± 0.1	95.2 ± 1.6	0.00 ± 0.09	3	7.5 ± 0.3	70.2 ± 8.3	0.00 ± 0.35	5
+ FLAG-RAMP3	10.3 ± 0.1	78.0 ± 2.5	0.05 ± 0.11	3	10.3 ± 0.0	93.4 ± 2.9	−0.17 ± 0.10	3	7.4 ± 0.2	77.6 ± 7.2	−0.07 ± 0.50	5
+ 2xFLAG-RAMP3	9.9 ± 0.1	66.1 ± 2.4∗	−0.38 ± 0.12	3	10.1 ± 0.2	91.6 ± 1.9	−0.34 ± 0.10	4	7.5 ± 0.2	89.9 ± 15.1	0.18 ± 0.30	5
Oxyntomodulin												
Receptor alone	8.0 ± 0.1	80.7 ± 1.2	0.00 ± 0.10	3	8.3 ± 0.0	94.8 ± 5.6	0.00 ± 0.26	3	6.9 ± 0.1	27.6 ± 4.8	0.00 ± 0.54	4
+ FLAG-RAMP3	7.9 ± 0.1	74.8 ± 2.5	−0.20 ± 0.10	3	8.3 ± 0.0	91.7 ± 3.3	−0.00 ± 0.20	3	6.6 ± 0.2	72.4 ± 20.4∗	0.03 ± 0.50	4
+ 2xFLAG-RAMP3	7.5 ± 0.0	63.3 ± 5.1∗	−0.56 ± 0.13 ∗	3	8.0 ± 0.3	92.1 ± 1.4	−0.29 ± 0.21	4	6.6 ± 0.3	70.8 ± 8.4∗	0.04 ± 0.51	4
GLP-1 (9–35)												
Receptor alone	NR			4	NR			4	NR			5
+ FLAG-RAMP3	NR			4	NR			4	NR			5
+ 2xFLAG-RAMP3	NR			4	NR			4	NR			5

NR designates where responses were too small to accurately calculate potency values. Mean values are recorded from n repeats performed in duplicate. Significant difference from receptor alone was calculated using a one-way ANOVA with Dunnett’s *post hoc* test (∗*p* < 0.05).

pEC_50_, *E*_max_, and LogRA values for cAMP accumulation and Ca^2+^ mobilization in HEK293T cells expressing Nluc–GLP-1R and increasing amounts of FLAG-RAMP3.

When increasing the stimulation period to 30 min, there was again a reduction in the potency of the response to GLP-1, with overexpression significantly reducing the pEC_50_ value (Fig. 2*C*, Table 1). Effects on other agonists were reduced, with significant differences no longer observed (Fig. 2*D*).

GLP-1R is a pleiotropic receptor, able to couple to G proteins other than Gα_s_ (7, 8, 23). In many studies, the receptor has been found to couple to the Gα_q/11_ family of G proteins, with GLP-1R activity also associated with increased intracellular Ca^2+^ concentrations (Ca^2+^)_i_ because of mobilization from intracellular stores. The effect of RAMP3 was therefore tested against (Ca^2+^)_i_ mobilization (Fig. 2, *E* and *F*, Table 1). FLAG-RAMP3 expression enhanced the (Ca^2+^)_i_ mobilization in response to GLP-1, increasing the potency of the response (Fig. 2*E*). The dataset was again extended to other agonists to determine any agonist-dependent effects. Again, GLP-1(9–36) was unable to elicit any (Ca^2+^)_i_ mobilization in the absence of RAMP3, meaning signaling parameters could not be calculated.

There was a significant trend for RAMP3 to increase maximal signaling in response to all agonists (*p* < 0.0001) (Fig. 2, *E* and *F*, Table 1). Expression in a 1:1 ratio significantly increased the maximal responses to exendin-4 (from 43.6 ± 5.30 to 71.61 ± 4.64% of 10 μM ionomycin, *p* < 0.05) and oxyntomodulin (from 21.7 ± 3.5 to 63.6 ± 10.6% of 10 μM ionomycin, *p* < 0.01). Overexpression also significantly increased the maximal response of these two agonists to 97.6 ± 6.8 and 52.3 ± 8.5, respectively (*p* < 0.0001, *p* < 0.05) as well as increasing the potency of the response to GLP-1 (from a pEC_50_ of 7.2 ± 0.2 to 8.1 ± 0.2, *p* < 0.05). There were no significant effects on the response to liraglutide, highlighting agonist dependency.

**Figure 3:**
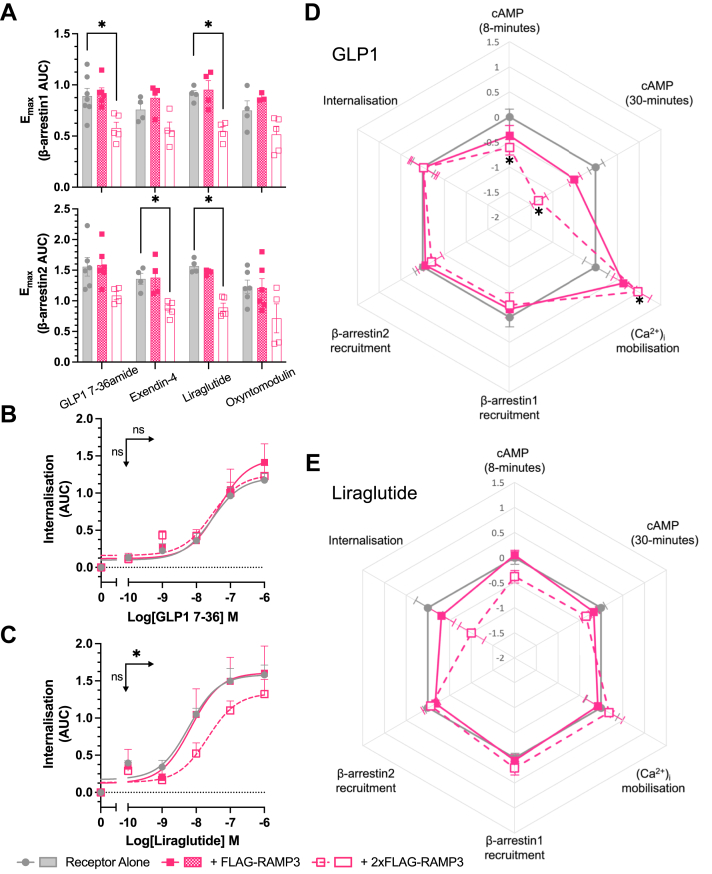
**Effects of desensitization are agonist dependent.***A*, maximal β-arrestin1 (*upper*) or β-arrestin2 (*lower*) recruitment to GLP-1R–Nluc, in response to GLP-1, exendin-4, liraglutide, or oxyntomodulin, n > 4. Internalization, as measured through a loss of BRET between GLP-1R–Nluc and plasma membrane marker RIT-Venus, in response to GLP-1 (*B*) or liraglutide (*C*), n > 3. *D* and *E*, LogRA values for second messenger signaling and receptor desensitization for GLP-1R, measured in HEK293T cells, in response to GLP-1 or liraglutide (n > 3). Responses are normalized to the response of GLP-1 at GLP-1R alone. Mean values of n experimental replicates are shown as ±SEM, and significance between RAMP conditions was determined through one-way ANOVA, ∗*p* < 0.05. *Horizontal arrows* indicate changes in potency (pEC_50_), and *vertical arrows* designate changes in efficacy (*E*_max_). BRET, bioluminescence resonance energy transfer; GLP-1, glucagon-like peptide-1; GLP-1R, GLP-1 receptor; RAMP, receptor activity–modifying protein.

As changes in signaling can result from altered receptor desensitization, we investigated the effects of RAMP3 expression on β-arrestin recruitment and internalization. This is especially pertinent when concerning RAMP3, as it is known to have effects on receptor internalization and trafficking for other class B1 GPCRs (24, 25, 26). Recruitment was reduced for all agonists except oxyntomodulin, although only when RAMP3 was expressed at a 2:1 ratio of the receptor (Fig. 3*A*). For GLP-1, exendin-4, and oxyntomodulin, there was no subsequent effect on internalization, consistent with the observation that GLP-1R internalization is β-arrestin independent (27) (shown for GLP-1 in Fig. 3*B*, Table 2). However, RAMP3 overexpression led to a significant decrease in the potency of internalization in response to liraglutide (Fig. 3*C*, Table 2).

**Table 2:** Influence of RAMP3 on GLP-1R desensitization and internalization—

	β-arrestin1				β-arrestin2				Internalization			
	pEC_50_	*E*_max_	LogRA	n	pEC_50_	*E*_max_	LogRA	n	pEC_50_	*E*_max_	LogRA	n
GLP-1 (7–36 amide)												
Receptor alone	7.4 ± 0.1	100.0 ± 5.6	0.00 ± 0.19	7	7.4 ± 0.1	100.0 ± 5.7	0.00 ± 0.19	6	7.5 ± 0.2	100.0 ± 7.1	0.00 ± 0.24	6
+ FLAG-RAMP3	7.2 ± 0.1	103.3 ± 4.6	−0.16 ± 0.16	6	7.3 ± 0.1	102.6 ± 5.9	−0.06 ± 0.19	6	7.4 ± 0.3	113.6 ± 13.9	−0.02 ± 0.33	4
+ 2xFLAG-RAMP3	7.3 ± 0.2	63.9 ± 6.0∗	−0.24 ± 0.25	4	7.3 ± 0.2	66.3 ± 5.6∗	−0.20 ± 0.23	4	7.5 ± 0.2	98.9 ± 8.7	−0.02 ± 0.29	4
Exendin-4												
Receptor alone	7.1 ± 0.1	87.4 ± 3.4	0.00 ± 0.11	4	7.3 ± 0.1	88.4 ± 2.7	0.00 ± 0.09	4	8.5 ± 0.3	96.0 ± 9.7	0.00 ± 0.44	3
+ FLAG-RAMP3	7.2 ± 0.1	99.2 ± 3.9	0.08 ± 0.11	4	7.3 ± 0.1	86.8 ± 3.3	0.03 ± 0.10	4	8.2 ± 0.3	106.1 ± 10.1	−0.27 ± 0.42	3
+ 2xFLAG-RAMP3	7.6 ± 0.2∗	58.7 ± 4.3∗	0.30 ± 0.19	4	7.6 ± 0.1	52.9 ± 3.3∗	0.06 ± 0.15	5	7.7 ± 0.3	116.3 ± 10.3	−0.75 ± 0.40	3
Liraglutide												
Receptor alone	7.0 ± 0.1	99.7 ± 4.0	0.00 ± 0.10	4	7.3 ± 0.1	98.6 ± 2.9	0.00 ± 0.09	4	8.5 ± 0.2	127.6 ± 7.0	0.00 ± 0.29	3
+ FLAG-RAMP3	7.1 ± 0.1	101.7 ± 5.8	0.05 ± 0.13	4	7.1 ± 0.0	92.3 ± 1.6	−0.17 ± 0.07	4	8.2 ± 0.2	124.4 ± 8.6	−0.31 ± 0.28	3
+ 2xFLAG-RAMP3	7.5 ± 0.1∗	58.1 ± 2.7∗	0.20 ± 0.13	4	7.5 ± 0.2	55.9 ± 3.9∗	−0.07 ± 0.17	5	7.6 ± 0.2∗	102.7 ± 8.0	−1.00 ± 0.29	3
Oxyntomodulin												
Receptor alone	5.7 ± 0.1	79.2 ± 4.8	0.00 ± 0.13	5	5.9 ± 0.1	80.7 ± 3.4	0.00 ± 0.10	6	6.5 ± 0.3	93.4 ± 13.1	0.00 ± 0.40	5
+ FLAG-RAMP3	5.8 ± 0.1	88.1 ± 6.1	0.14 ± 0.14	4	6.0 ± 0.1	78.6 ± 3.0	0.05 ± 0.10	6	6.8 ± 0.2	89.5 ± 9.5	0.26 ± 0.34	5
+ 2xFLAG-RAMP3	6.0 ± 0.2	61.9 ± 8.1	0.14 ± 0.22	6	5.5 ± 0.1∗	97.3 ± 6.4∗	−0.29 ± 0.11	5	7.3 ± 0.3	75.5 ± 8.0	0.65 ± 0.39	5
GLP-1 (9–36)												
Receptor alone	NR			4	NR			4	NR			3
+ FLAG-RAMP3	NR			4	NR			4	NR			3
+ 2xFLAG-RAMP3	NR			5	NR			4	NR			3

NR designates where responses were too small to accurately calculate potency values. Mean values are reported from n repeats in performed duplicate. Significant difference from receptor alone was calculated using a one-way ANOVA with Dunnett’s *post hoc* test (∗*p* < 0.05).

pEC_50_, *E*_max_, and LogRA values for β-arrestin1 and β-arrestin2 recruitment to GLP-1R–Nluc, expressed in HEK293T cells.

In order to make comparisons across pathways and agonists, logarithmic values of relative activity (LogRA) were calculated as metrics of bias for second messenger signaling assays (cAMP and [Ca^2+^]_i_ mobilization) and desensitization (Tables 1 and 2). The effects of RAMPs overall varied when looking at different peptides. GLP-1 showed a decrease in cAMP accumulation and an increase in (Ca^2+^)_i_ mobilization, with little effect on desensitization (Fig. 3*D*). However, liraglutide displayed lesser effects on second messenger signaling but a greater reduction in internalization (Fig. 3*E*).

### RAMP3 expression influences G protein coupling

To determine the underlying changes in G protein coupling associated with this biased second messenger production, the TRUPATH G protein biosensor platform was used (28). Three major families were examined, as they are closely linked with changes in cAMP accumulation and (Ca^2+^)_i_ mobilization: Gα_s_, Gα_i/o_, and Gα_q/11_. There are reports that the TRUPATH system struggles to detect Gα_q_ coupling at classically Gα_s_-coupled receptors (28, 29). We therefore tested the WT Gα_q_, and the R183Q mutant (Gα_q_(Q)), which displays increased constitutive and agonist-induced activity (30), in response to all four full agonists. No quantifiable response was observed using WT Gα_q_, as has been observed previously (29).

**Figure 4:**
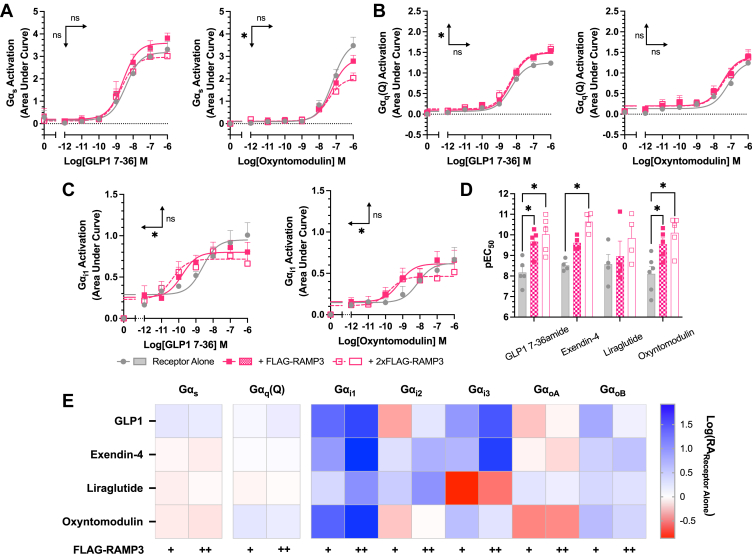
**RAMP3 alters G protein coupling of GLP-1R differently depending on the peptide agonist.** G protein coupling measured using the TRUPATH biosensor platform in HEK293T cells transfected with GLP-1R (*A*), Gα_s_, (*B*) Gα_q_(R183Q), and (*C*) Gα_i1_ shown in response to GLP-1 and oxyntomodulin. *D*, pEC_50_ values for Gα_i1_ coupling in response to all agonists. *E*, LogRA values for all Gα subunits tested, relative to receptor alone. Mean values are shown of n experimental replicates, ±SEM, where n > 4. Significance between RAMP conditions determined through one-way ANOVA, ∗*p* < 0.05. *Horizontal arrows* indicate changes in potency (pEC_50_), and *vertical arrows* designate changes in efficacy (*E*_max_). GLP-1, glucagon-like peptide-1; GLP-1R, GLP-1 receptor; HEK293T, human embryonic kidney 293T cell line; RAMP, receptor activity–modifying protein.

Whilst no effect was observed for GLP-1, exendin-4, or liraglutide, there was a significant reduction in the maximal Gα_s_ dissociation elicited by oxyntomodulin when RAMP3 was expressed (reduced to 58.9% ± 5.8 of receptor alone) (Fig. 4*A*, Table S1). There was no effect on the Gα_q_(Q) mutant response to exendin-4, liraglutide, or oxyntomodulin, but RAMP3 did significantly increase the maximal dissociation elicited by GLP-1 (to 119 ± 5.5% of receptor alone) (Fig. 4*B*, Table S1).

Whilst the effects on Gα_s_ and Gα_q_ provide some insights as to how RAMP3 exerts its effects on second messenger signaling, it is not conclusive. Linked with both a reduction in cAMP and an increase in (Ca^2+^)_i_ is Gα_i/o_. GLP-1R coupling to this family of G proteins has been observed previously (7, 8), so the effects of RAMP3 were studied across the family. GLP-1R showed little coupling to Gα_z_, which was unchanged by RAMP3 expression. However, for other members of the family, there was a significant increase in the potency of coupling as increasing concentrations of RAMP3 were expressed (Gα_i1_ shown in Fig. 4, *C* and *D*, Table S1).

To better enable comparisons between the different Gα subunits, LogRA values were calculated, relative to the response to the receptor alone (Fig. 4*E*, Table S1). The changes at Gα_s_ and Gα_q_(Q) were slight, mirroring the effects when only looking at the potency (pEC_50_) and efficacy (*E*_max_) parameters. Larger changes were observed in Gα_i/o_ coupling, with significant decreases in the LogRA for Gα_i1_ when RAMP3 was overexpressed in cells stimulated with exendin-4 and oxyntomodulin.

### Signaling in an insulin-secreting cell line

**Figure 5:**
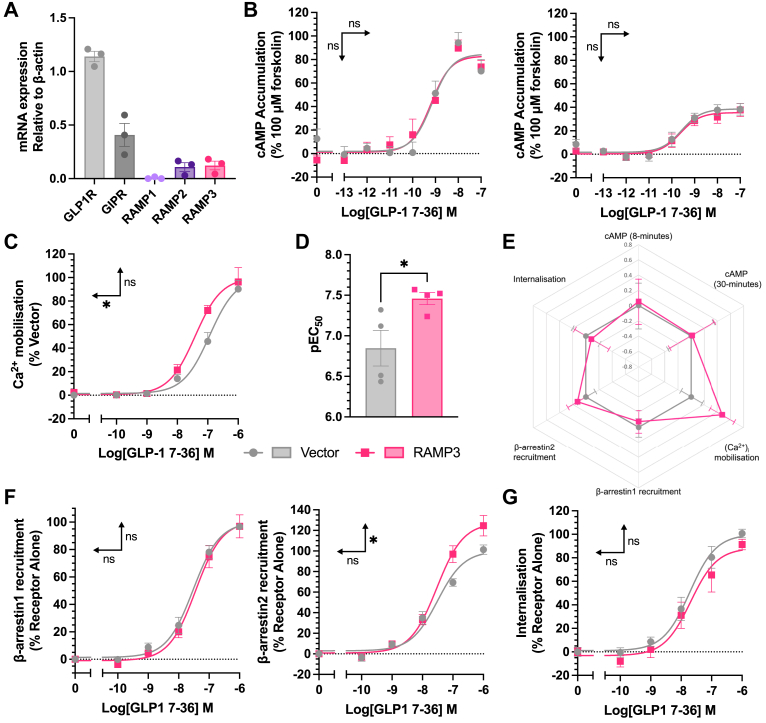
**Selective enhancement of Ca^2+^ mobilization by increased RAMP3 expression.***A*, mRNA expression of incretin hormone receptors and RAMPs in INS-1 832/3 cells, relative to β-actin (n = 3). *B*, cAMP accumulation in response to 8- or 30-min stimulation with GLP-1, in INS-1 832/3 cells transfected with vector or RAMP3 (n > 4). Responses are expressed as percentage of 100 μM forskolin (*C*) Ca^2+^ mobilization in response to stimulation with GLP-1, in INS-1 832/3 cells transfected with vector or RAMP3, with pEC_50_ values plotted in (*D*), n = 4. Responses are normalized to 10 μM ionomycin. *E*, radar plot of LogRA values for second messenger accumulation and receptor desensitization, relative to receptor alone. *F*, β-arrestin1 (*left*) or β-arrestin2 (*right*) recruitment to GLP-1R–Nluc, transiently transfected into INS-1 832/3 cells, n > 3. *G*, internalization of GLP-1R–Nluc in INS-1 832/3 cells, as determined through a loss of BRET with RIT-Venus (n = 4). Responses are normalized to vector-transfected cells. Mean values of n experimental replicates are shown ±SEM, and significance between RAMP conditions was determined through Student’s *t* test, ∗*p* < 0.05. *Horizontal arrows* indicate changes in potency (pEC_50_), and *vertical arrows* designate changes in efficacy (*E*_max_). BRET, bioluminescence resonance energy transfer; GLP-1, glucagon-like peptide-1; GLP-1R, GLP-1 receptor; RAMP, receptor activity–modifying protein.

GLP-1R signaling is inherently linked with insulin secretion, as receptor activation significantly enhances GSIS. This is linked to both cAMP accumulation and (Ca^2+^)_i_ mobilization, enhancing sensitivity of the insulin secretion machinery, with β-arrestin recruitment and internalization both positively and negatively implicated in GSIS (31, 32). To confirm the changes observed in human embryonic kidney 293T (HEK293T) cells are applicable to a more physiological setting, the aforementioned signaling assays were repeated in INS-1 832/3 cells, a rat insulinoma cell line. These cells endogenously express the GLP-1R, alongside low levels of RAMP2 and RAMP3 (Fig. 5*A*). RAMP3 was transfected into these cells, maintaining the endogenous expression of GLP-1R to look at the second messenger signaling. Assaying at 8- and 30-min stimulation, no effect was observed on cAMP accumulation in response to GLP-1 when RAMP3 was expressed (Fig. 5*B*, Table 3). However, as observed in HEK293T cells, RAMP3 did increase the potency of (Ca^2+^)_i_ mobilization (from a pEC_50_ of 6.94 ± 0.11 to 7.41 ± 0.14) (Fig. 5, *C* and *D*, Table 3). When calculating LogRA values, there is bias toward (Ca^2+^)_i_ mobilization, reflecting this enhanced potency (Fig. 5*E*), although the effect was not significant.

**Table 3:** Second messenger signaling and desensitization in a pancreatic β-cell model is influenced by RAMP expression—

	Vector				RAMP3			
	pEC50	*E*_max_	LogRA	n	pEC50	*E*_max_	LogRA	n
cAMP (8 min)	9.2 ± 0.3	87.3 ± 8.1	0.00 ± 0.30	3	9.4 ± 0.2	83.6 ± 6.7	0.05 ± 0.30	4
cAMP (30 min)	9.5 ± 0.3	47.9 ± 3.9	0.00 ± 0.35	6	9.4 ± 0.3	44.0 ± 4.1	0.01 ± 0.34	6
Ca^2+^ mobilization	6.9 ± 0.1	100.0 ± 6.6	0.00 ± 0.16	4	7.4 ± 0.1∗	99.9 ± 6.1	0.48 ± 0.18	4
β-arrestin1 recruitment	7.5 ± 0.1	100.0 ± 3.6	0.00 ± 0.12	3	7.4 ± 0.1	100.8 ± 5.4	−0.08 ± 0.15	3
β-arrestin2 recruitment	7.5 ± 0.1	100.0 ± 4.0	0.00 ± 0.14	7	7.5 ± 0.1	127.3 ± 6.0∗	0.13 ± 0.15	3
Internalization	7.7 ± 0.1	100.0 ± 5.9	0.00 ± 0.20	4	7.7 ± 0.2	88.3 ± 8.1	−0.08 ± 0.26	4

Values are mean from n repeats performed in duplicate, ±SEM. Significance was determined using a Student’s *t* test (∗*p* < 0.05).

pEC_50_, *E*_max_, and LogRA values for cAMP accumulation and Ca^2+^ mobilization, β-arrestin recruitment, and internalization in INS-183 2/3 cells. cAMP accumulation and Ca^2+^ mobilization were measured in cells transfected with vector or RAMP3, whereas β-arrestin recruitment and internalization used cells transfected with GLP-1R–Nluc, and β-arrestin–YFP or RIT-Venus, alongside vector or RAMP3.

When looking at β-arrestin recruitment or internalization, INS-1 832/3 cells without endogenous GLP-1R expression (33) were used to avoid any potential interference. Cells were transfected with GLP-1R–Nluc and either vector or RAMP3, and β-arrestin1/2–YFP for β-arrestin recruitment, or RIT-Venus for internalization. RAMP3 had no effect on β-arrestin1 recruitment but increased maximal β-arrestin2 recruitment in this cell line (Fig. 5*F*). This shows a system dependency to the effects of RAMP3, with the opposite effect observed in HEK293T cells. As in HEK293T cells, there was no effect on internalization (Fig. 5*G*, Table 3).

### Insulin secretion is enhanced by RAMP3

**Figure 6:**
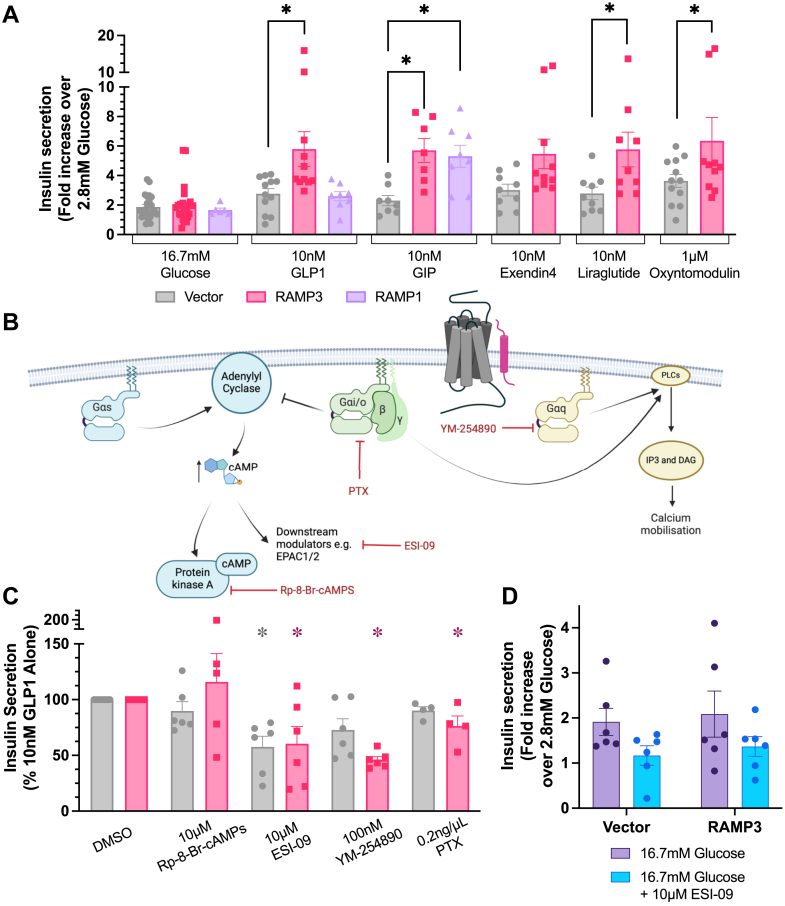
**RAMP3 enhances incretin-mediated insulin secretion, dependent on (Ca^2+^)_i_ mobilization.***A*, glucose-stimulated insulin secretion in INS-1 832/3 cells, transfected with vector, RAMP3, or RAMP1 and costimulated with 16.7 mM glucose and GLP-1, GIP, exendin-4, liraglutide, or oxyntomodulin. Results are expressed as fold change over 2.8 mM glucose, n > 7. *B*, schematic illustrating the action of different small-molecule inhibitors of G protein signaling, and their effects on insulin secretion in INS-183 2/3 cells transfected with vector or RAMP3. *C*, insulin secretion in INS-183 2/3 cells transfected with vector or RAMP3 and stimulated with 10 nM GLP-1. Cells were pretreated and costimulated with inhibitors of G protein signaling. Responses are normalized to cells treated with 10 nM GLP-1 and 0.1% DMSO, which acted as a vehicle control, n > 4. *D*, insulin secretion in response to 16.7 mM glucose, either in the absence or in the presence of 10 μM ESI-09, n = 6. Mean values of n experimental replicates are shown ±SEM, significance between RAMP conditions was determined through one-way ANOVA (GIPR) or two-way ANOVA (GLP-1R), or Kruskal–Wallis test (GLP-1R inhibitor dataset) ∗*p* < 0.05. *Horizontal arrows* indicate changes in potency (pEC_50_), and *vertical arrows* designate changes in efficacy (*E*_max_). (Ca^2+^)_i_, intracellular Ca^2+^ concentration; DMSO, dimethyl sulfoxide; GIP, glucose-dependent insulinotropic polypeptide; GIPR, GIP receptor; GLP-1, glucagon-like peptide-1; GLP-1R, GLP-1 receptor; RAMP, receptor activity–modifying protein.

INS-1 832/3 cells overexpressing RAMP3 displayed significantly greater insulin secretion in response to 10 nM GLP-1 than those expressing vector; GLP-1 increased insulin secretion 2.36 ± 0.30-fold over 2.8 mM glucose compared with 3.94 ± 0.33 when RAMP3 is overexpressed (Fig. 6*A*). This was not observed when cells were transfected with RAMP1, although both RAMP1 and RAMP3 increased insulin secretion in response to 10 nM GIP, whose receptor interacts with both proteins (17) (Fig. 6*A*). The effect of RAMP3 was confirmed for the other GLP-1R agonists, whose signaling has also been affected by RAMP3 expression, although the increase was not significant for exendin-4.

To determine how this increase in insulin secretion occurs, inhibitors of different G protein–mediated signaling pathways were employed (schematic shown in Fig. 6*B*). Data have been normalized to the response to GLP-1 in the absence of an inhibitor.

As no specific inhibitor of Gα_s_ is available, inhibitors of proteins whose signaling is downstream of cAMP accumulation were used. Inhibition of PKA with Rp-8-Br-cAMPs had no effect on insulin secretion, irrespective of the expression of RAMP3 (Fig. 6*C*). However, inhibition of exchange factor directly activated by cAMP1/2 (EPAC1/2) with the dual inhibitor ESI-09 significantly decreased GLP-1-mediated potentiation of GSIS in both vector- and RAMP3-transfected cells (Fig. 6*C*). However, a reduction was also observed in the response to 16.7 mM glucose alone (Fig. 6*D*).

Inhibition of Gα_q/11_ activation with 100 nM YM-254890 or Gα_i/o_ signalling with pertussis toxin (PTX) decreased insulin secretion significantly only when RAMP3 was overexpressed. It is therefore suggested that RAMP3 enhances Gα_q/11_ and Gα_i/o_ activation, enhancing (Ca^2+^)_I_ mobilization and GSIS.

### RAMP3 is important for GLP-1R-mediated insulin secretion

**Figure 7:**
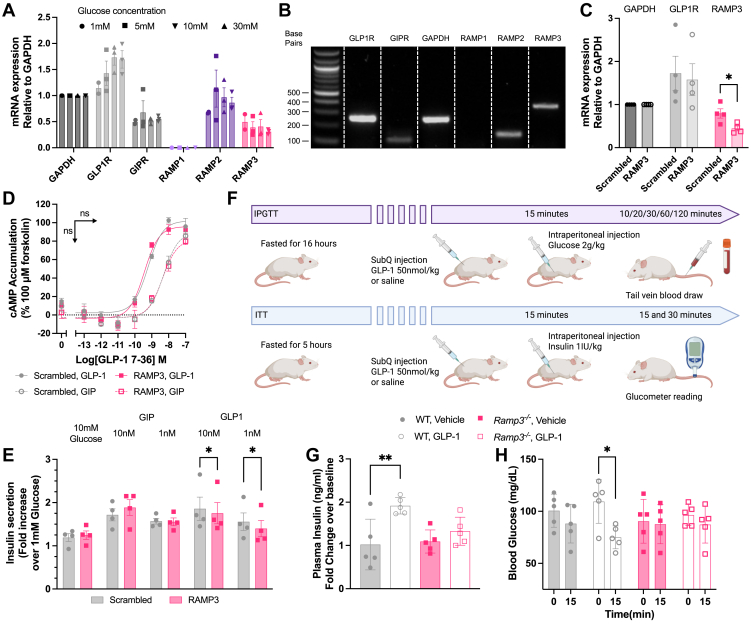
**Reducing RAMP3 expression decreases sensitivity to GLP-1 in murine models.***A*, mRNA expression of GLP-1R, GIPR, RAMP1, RAMP2, and RAMP3 in Min6 B1 cells, cultured in varied glucose concentrations (n = 3), with example gel shown in (*B*). Data are normalized to GAPDH. *C*, mRNA expression of GLP-1R and RAMP3, relative to GAPDH, in Min6 B1 cells treated with scrambled RAMP3 siRNA (n = 4). *D*, cAMP accumulation in siRNA-treated Min6 B1 cells, in response to 8-min stimulation with GLP-1 (*closed symbols*) or GIP (*open symbols*), n = 4. *E*, insulin secretion in response to 10 mM glucose, with or without GIP or GLP-1, in Min6 B1 cells treated with scrambled or RAMP3 siRNA, n = 4. *F*, schematic for treatment of WT and Ramp3^−/−^ mice for IPGTT and ITT. *G*, plasma insulin for IPGTT WT and Ramp3^−/−^ mice, expressed a fold change over baseline responses (n = 5). *H*, blood glucose concentrations of WT and Ramp3^−/−^ mice, following ITT. Measured at baseline (0) and after 15 min, n = 5. Mean values of n experimental replicates are shown ±SEM (or SD for biological replicates in *G* and *H*). Significance was determined through Student’s *t* test for RAMP3 knockdown or matching two-way ANOVA (*E* and *H*) or one-way ANOVA (*G*), with Tukey’s test for multiple comparisons. GIP, glucose-dependent insulinotropic polypeptide; GLP-1, glucagon-like peptide-1; GLP-1R, GLP-1 receptor; HEK293T, human embryonic kidney 293T cell line; IPGTT, intraperitoneal glucose tolerance test; ITT, insulin tolerance test; RAMP, receptor activity–modifying protein.

As these experiments focused on the influence of RAMP3 overexpression on GLP-1R pharmacology, we next investigated the effect of RAMP3 knockdown in circumstances where it is endogenously expressed. Whilst INS-1 832/3 cells express low levels of RAMP3, Min6 B1, a mouse islet cell model, was shown to express both RAMP2 and RAMP3. This expression was unaffected by glucose culture conditions, although the GLP-1R expression correlated with increasing glucose concentration (Fig. 7*A*, an example gel is shown in Fig. 7*B*). RAMP3 mRNA was reduced using siRNA treatment to around 50% of cells treated with control, scrambled siRNA (Fig. 7*C*). There was no effect on GLP-1R expression or cAMP accumulation mediated by the GLP-1R or GIPR, following 8-min stimulation with GLP-1 or GIP, respectively (Fig. 7*D*). Insulin secretion mediated by glucose with or without 10 nM or 1 nM GIP was unaffected by RAMP3 knockdown, but the response to 10 nM and 1 nM GLP-1 was attenuated, although the effect was small (Fig. 1*E*). This is likely because of the relatively low RAMP3 expression and only partial knockdown in expression.

To assess how these effects translate into more physiologically relevant systems, we moved to a global Ramp3 gene deletion mouse model (15). This allowed for measurement of the intraperitoneal glucose tolerance test (IPGTT) and insulin tolerance test (ITT) (Fig. 7*F*). In WT mice, GLP-1 increased insulin secretion 15 min after administration of glucose. This response was lost in *Ramp3*^*−/−*^ mice. GLP-1 also potentiated the effects of insulin on blood glucose, an effect that too was lost in *Ramp3*^*−/−*^ mice (Fig. 7*H*). Together, these data demonstrate the importance of RAMP3 for the functional, rapid response of GLP-1 signaling in the control of glucose homeostasis.

## Discussion

Insulin secretion following ingestion of food high in carbohydrates is greatly potentiated by signaling of incretin hormones. Manipulation of the signaling of the incretins is therefore a valuable strategy for treating diabetes. However, development of therapeutics targeting the GLP-1R has been made difficult by the variability in the signaling of the receptor depending on the study. Part of this variability is attributed to the cell background in which the assay is performed and the different interacting proteins expressed therein. We therefore sought to identify any interactions with the RAMP family of proteins, which modulate signaling at many class B1 GPCRs, and determine any subsequent effects on signaling of exogenously and endogenously expressed receptors. We then further delved into the effect of these changes in signaling on GSIS.

GLP-1R was found to interact with RAMP3 but not the other members of the RAMP family of proteins. Previous studies have shown interactions with RAMP1 and RAMP2, although at a reduced level. However, these studies have used isolated lysates (18) or whole cell resonance energy transfer (17, 19). Neither technique discerns between interactions in different subcellular compartments and therefore does not confirm a functional receptor complex. Use of a cell-impermeable dye to irreversibly label SNAP-RAMP enables measurement of only interactions at the plasma membrane, suggesting interactions previously observed with RAMP1 and RAMP2 occurring in intracellular compartments. Furthermore, adaptation of the NanoBiT system for ligand binding determined it is only the GLP-1R–RAMP3 complex capable of agonist binding and not the other two RAMPs. This interaction had no effect on membrane expression of either GLP-1R or RAMP3 nor on binding of GLP-1 to the receptor. Unlike other RAMPs, RAMP3 is able to translocate to the plasma membrane in the absence of an interacting GPCR (12). It is therefore possible that it displays interactions with an increased number of GPCRs through proximity, as opposed to a specific interaction (26). However, increasing the expression of RAMP1 at the membrane through overexpression of CLR had no effect on the interaction observed.

RAMP3 expression did, however, modulate G protein signaling at the receptor. Consistent with previous observations (19), cAMP accumulation was decreased in the presence of RAMP3, especially at early time points. However, unlike previous reports, an increase in (Ca^2+^)_i_ mobilization was observed with increasing expression of RAMP3. These findings were validated by examining the effects on G protein coupling, using the TRUPATH biosensor platform. At individual agonists, small perturbations could be observed. Gα_s_ coupling in response to oxyntomodulin was increased by RAMP3, and Gα_q_ coupling was increased when cells were stimulated with GLP-1. However, for the remaining agonists, no effects were observed. This did not correlate with the second messenger signaling results, where effects were more widespread across the agonists. Gα_i/o_ coupling, whilst small, was increased by RAMP3 in response to all agonists tested. In correlation with the effects on cAMP production, β-arrestin recruitment was decreased by RAMP3 overexpression, although not for the weaker agonist oxyntomodulin. There were no subsequent effects on internalization for most agonists, but the response to liraglutide was reduced. The GLP-1R has been shown to internalize through multiple avenues, with no β-arrestin dependency (27); so a lack of correlation is not surprising.

In many of the assays studied, ligand-dependent effects were observed; for example, selective enhancement of different G proteins or differential effects on efficacy compared with potency. The agonists themselves have their own inherent signaling bias, with liraglutide displaying bias toward β-arrestin recruitment. This may then correlate with arrestin-dependent internalization not observed for the other agonists, explaining why RAMP3 only alters internalization in this case. It is thought that agonist bias stems from stabilization of distinct receptor conformations, promoting different G protein couplings (34). RAMPs can also alter GPCR conformational states (35), leading to different effects depending on the ligand. Without structural interrogation, it is difficult to ascertain how these agonist-dependent effects emerged. However, these differences were primarily the magnitude of the effect, with RAMP3 still generally moving GLP-1R toward (Ca^2+^)_i_ mobilization and insulin secretion in response to all agonists.

Insulin secretion is enhanced by overexpression of RAMP3. This was observed for all GLP-1R agonists tested but not high glucose alone. It is therefore unlikely the increase in RAMP3 expression itself directly increases insulin secretion. Furthermore, increasing RAMP1 expression (which does not interact with GLP-1R) had no effect on the potentiation of GSIS by GLP-1R but did enhance GIPR-mediated GSIS, consistent with the findings that reducing RAMP1 expression reduced glucose tolerance in mice (17).

GLP-1-mediated GSIS was unaffected by inhibition of PKA by Rp-8-Br-cAMPs in the presence or the absence of RAMP3. Previous studies have shown potentiation of GSIS to be both PKA dependent (36) and independent (37), with this study agreeing with the latter, although this might be due to reduced affinity of the compound for the rat kinases. Inhibition of EPAC1/2 with ESI-09 significantly reduced insulin secretion, although a nonsignificant reduction was also observed in cells stimulated with high glucose alone, suggesting importance in GSIS independent of the incretin hormones. EPAC1/2 has been previously shown to be important for cAMP-dependent potentiation of GSIS (38), which these data support. As cAMP accumulation was unaffected in the INS-1 832/3 cell line by the overexpression of RAMP3, it is not surprising that there was no interactive effect of RAMP3 when PKA or EPAC1/2 was inhibited. YM-254890 and PTX, inhibitors of Gα_q/11_ and Gα_i/o,_ respectively, however, only reduced insulin secretion in cells overexpressing RAMP3. This is consistent with the elevation in (Ca^2+^)_i_ mobilization when RAMP3 was overexpressed, dependent on enhanced Gα_i/o_ coupling. Gα_i/o_-mediated (Ca^2+^)_i_ mobilization has been shown to depend on active Gα_q/11_ (39), making the resolution of the respective contributions of the two G protein families difficult. However, the effect of YM-254890 was greater than PTX, suggesting a contribution from both families.

In murine models, endogenous RAMP3 was shown to be important, as a reduction in expression attenuated effects of GLP-1 on insulin secretion and glucose tolerance. As with the overexpression data for INS-1 832/3 cells, RAMP3 knockdown had no effect on cAMP accumulation. It is possible this difference is due to lower receptor expression or a differential effect on bias for the rodent receptors than the human. Species differences in signaling bias have been observed for other GPCRs associated with islet cell pharmacology (40), so it stands to reason this might be influencing the differences observed here. However, with over 90% sequence identity, it is more likely that the effects seen in the HEK293T cells are products of the receptor overexpression, as this has been noted as a factor in agonist bias (41). When expanded to an animal model with global deletion of *Ramp3*, this reduction in insulin secretion became more pronounced, with GLP-1 no longer able to elevate insulin secretion above vehicle-treated animals. This then led to an elevation in blood glucose concentration, again through a loss of response to GLP-1. *Ramp3* KO has previously been shown to increase the effect of GLP-1 on bodyweight lowering in mouse models, highlighting the preservation of a functional receptor within these animals (42) This also highlights the complexity of GLP-1R pharmacology, with tissue- and context-dependent effects of *Ramp3* KO; an elevation in one function but a loss of another. These differences may stem from the deletion of only *Ramp3* in this study compared with the dual *Ramp1/Ramp3* deletion described in the study by Leuthardt *et al.* or may relate to differences in GLP-1R–RAMP3 pharmacology in endocrine *versus* neuronal tissues. These results may therefore present an opportunity to further separate the role of the receptor in glucose homeostasis and appetite regulation.

Within this study, RAMP3 has been shown to influence agonist bias at the GLP-1R, shifting the receptor toward (Ca^2+^)_i_ mobilization, which enhances insulin secretion. As RAMP3 expression varies between tissues, it is possible that by directly targeting this complex, tissue-specific receptor activation could be facilitated. However, of more relevance is the consideration of RAMP expression when developing new GLP-1 mimetics or small-molecule agonists and allosteric modulators for the treatment of T2DM and obesity. Agonists that have reduced affinity for the RAMP3 complex over the receptor alone might show reduced therapeutic potential, which might not be detected when only looking at the GLP-1R alone. Furthermore, it is important to ensure that allosteric modulators do not disrupt the interaction, as is the case for the CLR–RAMP1 antagonist BIBN 4096 BS (43), as this could lead to a reduction in GLP-1R activity in cells expressing RAMP3.

## Experimental procedures

### Materials

GLP-1 (7–36 amide), oxyntomodulin, exendin-4, and GLP-1 (9–36 amide) were custom synthesized by Generon. Liraglutide was purchased from Cayman Chemicals, and GIP and GLP-1 (for *in vivo* experiments) were purchased from Bachem. Peptides were dissolved at 1 mM (or 10 mM in the case of GLP-1 (9–36 amide) in dimethyl sulfoxide (DMSO) or water with 0.1% bovine serum albumin (BSA) w/v for GIP and GLP-1 (9–36 amide). Forskolin was purchased from Cayman Chemicals and made up to 10 mM in DMSO, and ionomycin was purchased from Cayman Chemicals and made up to 10 mM in ethanol. YM-254890 (Wako Chemicals, Alpha Laboratories) was made up to 1 mM, and Rp-8-Br-cAMPs (Sigma–Aldrich) and ES109 (Bio-Techne) were stored at 10 mM in DMSO. PTX (Gibco, Thermo Scientific) was stored as a 0.1 mg/ml solution in water (w/v).

### DNA constructs

LgBiT-GLP-1R and Nluc–GLP-1R were made by PCR amplification of GLP-1R and restriction enzyme digest (Invitrogen, Thermo Scientific). This was then ligated into pcDNA3.1(+)sig-LgBiT or pcDNA3.1(+)sig-Nluc (where sig is the signal peptide of the murine serotonin 5-HT3R). GLP-1R–Nluc was made *via* the same process but was ligated into pcDNA3.1(−)-Nluc. SmBiT-RAMPs were generated by restriction enzyme digest of the RAMP with EcoRI and EcoRV (Invitrogen, Thermo Scientific), the product of which was then ligated into pcDNA3.1(+)-sigSmBiT. Sequences were confirmed by Sanger sequencing (University of Cambridge). RIT-Venus was donated by Luke Pattison (44), and β-arrestin1/2–YFP were donated by Duncan Mackie (29). Untagged RAMP1 and RAMP3 were purchased from cDNA.org. Use of Nluc-CLR, SNAP-RAMPs, and FLAG-RAMP3 has been described previously (17, 45). TRUPATH G protein constructs were a gift from Bryan Roth (Addgene kit #1000000163).

### Cell culture and transfection

Cos7 and HEK293T cells were grown in Dulbecco’s modified Eagle's medium (DMEM)/F12 Glutamax supplemented with 1% antibiotic–antimycotic (AA) solution and 10% fetal bovine serum (FBS) v/v. INS-1 832/3 and INS-1 832/3 GLP-1RKO cells, donated by Jacqueline Naylor (AstraZeneca), were grown in RPMI supplemented with 1% AA, 5% FBS, 10 mM Hepes, 50 μM β-mercaptophenol, and 1 mM sodium pyruvate. Min6 B1 cells (gifted by Dr Philippe Miyazaki, University of Osaka) were cultured in DMEM containing 4.5 g/l glucose, supplemented with 15% FBS, 1% penicillin–streptomycin, and 50 μM β-mercaptophenol. Cells were grown at 37 °C with 5% CO_2_. Transfection of cells with polyethyleneimine was conducted at a 6:1 v/w ratio of polyethyleneimine:DNA in 150 mM NaCl. Transfection with FuGENE HD (Promega) was carried out according to the manufacturer’s guidance, using a 3:1 v/w ratio of FuGENE HD:DNA. Transfection of INS-1 cell lines was carried out with Lipofectamine 2000 according to the manufacturer’s instructions, using a 3:1 ratio v/w of Lipofectamine 2000:DNA. For siRNA knockdown of RAMP3, Min6 B1 cells were treated with 20 nM siRNA for 48 h, using Lipofectamine 3000 at a 3:1 v/w ratio. Control scrambled siRNA was purchased from Invitrogen (negative control siRNA 1). ON-TARGETplus SMARTpool siRNA targeting mouse RAMP3 was purchased from Horizon Discovery (Revvity), containing sequences: 5′-GGUGCAACCUGUCGGAGUU-3′, 5′-GUAUGCGGCUGCAACGAGA-3′, 5′-GCACCGAGAUGGAGACCAA-3′, and 5′-GAAGUACUCAUCCCACUGA-3′.

### Receptor–RAMP interactions and binding

Experiments were adapted from those detailed by Harris *et al.* (17). Cos7 cells were seeded at 15,000 cells per well of a 0.01% poly-l-lysine (PLL)-coated 96-well white culture plate and incubated overnight. Cells were transfected with 50 ng Nluc-GPCR and increasing amounts of SNAP-RAMP. After 24 h, growth media were replaced with serum-free DMEM/F12 containing 200 nmol of SNAP-Surface Alexa Fluor 488 and incubated for 30 min. Cells were washed and incubated in KREBs (126 mM NaCl, 2.5 mM KCl, 25 mM NaHCO_3_, 1.2 mM NaH_2_PO_4_, 1.2 mM MgCl_2_, and 2.5 mM CaCl_2_) containing 0.1% BSA w/v and 0.1% Nano-Glo Cell Assay Substrate (Promega) v/v and read using a Mithras LB microplate reader at 460 and 515 nm. The ratio at the 10-min time point was used for further analysis. For NanoBiT interaction assays, HEK293T cells expressing increasing amounts of SmBiT-RAMP and 100 ng LgBiT-GPCR were incubated in KREBs containing 0.2% NanoGlo Cell Assay Substrate v/v. The 10-min time point was used to construct saturation curves.

The Tag-lite GLP-1R fluorescent probe (Cisbio), based on exendin-4, was used for binding assays (Ex-4-Red). LgBiT-GLP-1R and SmBiT-RAMP or Nluc–GLP-1R and FLAG-RAMP3 were coexpressed in HEK293T cells at a 1:1 or 1:2 ratio. Cells were incubated in KREBs containing 0.1% or 0.2% v/v NanoGlo for 5 min. Immediately before reading, Ex-4-Red in the presence of GLP-1, 10 μM exendin-9, or vehicle was added. Where injection was used, 50 μl of 1 μM GLP-1 in KREBs supplemented with 0.1% BSA (w/v) and 0.1% Nano-Glo (v/v) was injected robotically at 20 min into a well containing 50 μl KREBs supplemented with 0.1% BSA (w/v) and 0.1% Nano-Glo (v/v) and 4 nM Ex4-Red. Plates were read using a Mithras LB 940 multimode microplate reader, using 460 nm and 610 nm long-pass emission filters at 30 s intervals.

### Flow cytometry to measure GPCR and RAMP surface expression

For GLP-1R surface expression, HEK293T cells were transfected with Nluc–GLP-1R and FLAG-RAMP3 at a 1:1 or 1:2 ratio. After 48 h, 300,000 cells were washed twice with fluorescence-activated cell sorting buffer (PBS supplemented with 1% BSA and 0.03% sodium azide) before and after incubation with rabbit anti-Nluc polyclonal antibody, diluted 1/100 (Promega) for 1 h at room temperature in the dark. Samples were analyzed using a BD Accuri C6 flow cytometer (excitation λ = 633 nm and emission λ = 660 nm). Data were normalized to the median allophycocyanin (APC) intensity of cells transfected with pcDNA3.1 as 0% and Nluc–GLP-1R + pcDNA3.1 as 100%. For RAMP cell surface expression, HEK293T cells were transfected with Nluc–GLP-1R or HA-CLR and FLAG-RAMP1/2/3 at a 1:1 ratio. After 48 h, 300,000 cells were washed twice with fluorescence-activated cell sorting buffer (PBS supplemented with 1% BSA and 0.03% sodium azide) before and after incubation with rat APC–conjugated anti-FLAG monoclonal antibody for 1 h at room temperature in the dark. Data were normalized to the median APC intensity of cells transfected with pcDNA3.1 as 0% and HA-CLR + FLAG-RAMP2 as 100%.

### Reverse transcriptase PCR to measure endogenous GPCR and RAMP expression

For studies assessing the effects of glucose culture conditions, Min6 B1 cells were cultured in glucose-free DMEM, supplemented with 15% FBS, 1% penicillin–streptomycin, 50 μM β-mercaptophenol, and 1 mM, 5 mM, 10 mM, or 30 mM glucose for 4 days prior to harvest. RNA was extracted from INS-1 832/3 and Min6 B1 cells using RNeasy mini kit (Qiagen) as per the manufacturer’s protocol. Complementary DNA was generated using the QuantiTect reverse transcription kit (Qiagen) following the manufacturer’s instructions with minus reverse transcriptase negative controls performed simultaneously. PCR amplification was performed as previously described (20, 46) using the following gene-specific primers: rat β-actin, forward (5′-CCGCGAGTACAACCTTCTTG-3′) and reverse (5′-CAGTTGGTGACAATGCCGTG-3′); rat GLP-1R, forward (5′-GGGCTCCTCTCGTATCAGGA-3′) and reverse (5′-GTGAACAGCTTGACGAAGCG-3′); rat GIPR, forward (5′-AGGTGGTATTTGCTCCCGTG-3′) and reverse (5′-AGGGGTCCCTTTACCTAGCA-3′); rat RAMP1, forward (5′-GATGTGAGGACAGGAACCAGA-3′) and reverse (5′-TGGTCTTTCCCCAGTCACAC-3′); rat RAMP2, forward (5′-CTCCGGAGTCCCTGAATCAA-3′) and reverse (5′-TCCAGTTGCACCAGTCCTTG-3′); rat RAMP3 forward (5′-ACAAACATCGTGGGCTGCTA-3′) and reverse (5′-CCACGGTCAACAAGACTGGA-3′); mouse GAPDH forward (5′-CAGGAGAGTGTTTCCTCGTCC-3′) and reverse (5′-GATGGGCTTCCCGTTGATGA-3′); mouse GLP-1R forward (5′-ACTCTCATCCCCCTTCTGGG-3′) and reverse (5′-GGACACTTGAGGGGCTTCAT-3′); mouse GIPR forward (5′-CGAGTGGCCAGAGTTTCCAT-3′) and reverse (5′-TCTGCCCCTCAGAGTCTGTC-3′); mouse RAMP1 forward (5′-AGCCGCTTCAAGGAGAACAT-3′) and reverse (5′-CGAAACTGCTTCCTGCAAACT-3′); mouse RAMP2 forward (5′-TGAGGACAGCCTTGTGTCAA-3′) and reverse (5′-GGTCGCTGTAATGCCTGCTA-3′); mouse RAMP3 forward (5′-AGTACTTCAGAGCCTAGAGGTGA-3′) and reverse (5′-ATAGCCACAGTCAGCACGAC-3′). Products were resolved on a 2% agarose gel and imaged using an iBright imager (Invitrogen). Band intensity was analyzed using Fiji (ImageJ) and normalized to the β-actin (INS-1 832/3) or GAPDH (Min6 B1).

### cAMP accumulation

HEK293T cells and INS-1 832/2 cells were cultured as before and transfected 24 h before assaying, with Nluc–GLP-1R and pcDNA3.1 and/or FLAG-RAMP3 (HEK293T) or 48 h before with pcDNA3.1 or RAMP3 (INS-1). Min6 B1 cells were seeded for 48 h following siRNA treatment. On the day of the assay, cells were dissociated into single-cell suspension with trypsin–EDTA (0.05%) and resuspended in PBS with 0.1% BSA w/v in the presence (INS-1 832/3 and Min6 B1) or the absence (HEK293T) of 500 μM IBMX. Cells were plated at 1000 (HEK293T), 2000 (INS-1 832/3), or 500 (Min6 B1) cells per well of a 384-well OptiPlate (PerkinElmer) in 5 μl aliquots. Cells were stimulated for a given period, and then the cAMP accumulation was measured using the LANCE *ultra* cAMP detection kit as per the manufacturers’ instructions. Data were normalized to 100 μM forskolin.

### Calcium mobilization assay

HEK293T cells were transfected with 100 ng Nluc–GLP-1R and 200 ng of pcDNA3.1 or FLAG-RAMP3 or 100 ng of pcDNA3.1 and FLAG-RAMP3 per 100 μl cells and seeded in a 0.01% PLL-coated black-walled clear-bottom 96-well plate. About 48 h later, media were removed, and cells were incubated in 10 μM Fluor4-AM dye containing 2.5 mM probenecid for 1 h at room temperature. Cells were washed, and the dye was replaced with Hank’s balanced salt solution (Lonza) without Ca^2+^ containing 0.1% BSA w/v. Ligands were added robotically using a BD Pathway 855 high-intensity bioimager, and images were taken at 0.5 s intervals for 120 s. The response to vehicle was taken to be 0%, and the response to 10 μM ionomycin was used as 100%. For HEK293T cells, the maximal fluorescence was used for further analysis. For INS-1 832/3 cells, the area under the curve for the first 120 s after ligand addition was used.

### G protein dissociation

G protein dissociation assays were performed using the TRUPATH biosensor platform, as described (28). HEK293T cells were transfected with GLP-1R, Gα, Gγ, Gβ, and pcDNA3.1 or FLAG-RAMP3, all in equal quantities, and incubated overnight. Cells were then seeded on white 96-well 0.01% PLL-coated plates in minimum essential media supplemented with 1% AA and 2% FBS at 50,000 cells per well and cultured overnight. Cells were washed with KREBs with 0.1% BSA and incubated in buffer containing 5 μM coelenterazine 400a for 5 min. Ligands were then added, and plates were read using the PHERAstar microplate reader using the BRET2 module, at 60-s intervals. The BRET2 ratio was calculated (λ_515_/λ_400_), and the total dissociation over a 30-min period was used to generate dose–response curves. Data were normalized to the response of the receptor alone to GLP-1 at each Gα protein.

### β-arrestin recruitment assays

β-arrestin recruitment assays were adapted from those previously described (22, 47). Cells were transfected with GLP-1R–Nluc, β-arrestin–YFP, and either pcDNA3.1 or FLAG-RAMP3 in a 1:5:2 ratio and incubated overnight. HEK293T cells were then reseeded onto a 0.01% PLL-coated white 96-well culture plate at 50,000 cells per well. INS-1 832/3 cells were reseeded at 100,000 cells per well in growth media. Stimulation buffer for HEK293T cells was KREBs containing 0.1% BSA, whereas KREBs-Ringer (2.6 mM CaCl_2_, 98.5 mM NaCl, 4 mM KCl, 1.2 mM KH_2_PO_4_, 1.2 mM MgSO_4_, 20 mM Hepes, 25.9 mM NaHCO_3_, pH 7.4) with 0.1% BSA was used for INS-1 832/3 cells. Cells were washed before being incubated in stimulation buffer with 0.1% Nano-Glo Cell Assay substrate for 5 min. Ligand was then added, and plates were immediately read using a Mithras LB 940 microplate reader, at 460 nm and 530 nm, for 1 h at 60 s intervals. Data were normalized to the response of the receptor alone to GLP-1 at each arrestin.

### Measurement of receptor internalization

Experiments were performed as previously described (47). Briefly, HEK293T or INS-1 832/3 GLP-1R KO cells were transfected with GLP–1R-Nluc, RIT-Venus, and either pcDNA3.1 or FLAG-RAMP3 in a 1:5:2 ratio and incubated overnight. HEK293T cells were then reseeded onto a 0.01% PLL-coated white 96-well culture plate at 50,000 cells per well, in MEM media supplemented with 1% AA and 2% FBS. INS-1 832/3 cells were reseeded at 100,000 cells per well in growth media. Stimulation buffer for HEK293T cells was KREBs containing 0.1% BSA, whereas KREBs-Ringer (2.6 mM CaCl_2_, 98.5 mM NaCl, 4 mM KCl, 1.2 mM KH_2_PO_4_, 1.2 mM MgSO_4_, 20 mM Hepes, 25.9 mM NaHCO_3_, pH 7.4) with 0.1% BSA was used for INS-1 832/3 cells. Cells were washed with stimulation buffer before being incubated in stimulation buffer with 0.1% Nano-Glo Cell Assay substrate for 5 min. Ligand was then added, and plates were immediately read using a Mithras LB 940 microplate reader, at 460 nm and 530 nm, for 1 h at 60 s intervals. Data were normalized to the response of GLP-1R alone to GLP-1.

### Insulin secretion

INS-1 832/3 cells were transfected with pcDNA3.1 or RAMP3 and seeded at 75,000 cells per well of a 0.01% PLL-coated clear 96-well plate. Min6 B1 cells were seeded at 50,000 cells per well following siRNA treatment. For experiments using PTX, after 24 h, PTX was added to a final concentration of 0.2 ng/μl and maintained throughout the assay. About 48 h after transfection, the cells were glucose starved in RPMI media without glucose (INS-1 832/3) or DMEM with 1 g/l glucose (Min6 B1) for 3 h. Cells were washed and incubated at 37 °C for 1 h in stimulation buffer (KREBs-Ringer with 0.1% BSA) containing 2.8 mM (INS-1 832/3) or 3 mM (Min6 B1) glucose before being stimulated for 1 h. Where signaling inhibitors were used, cells were incubated with the inhibitor or vehicle control for 30 min prior to stimulation. The supernatant was removed, and the cells were incubated for 5 min in stimulation buffer with 1% Triton X-100 (v/v). Supernatant and lysed cell solution were centrifuged at 2000 rpm for 5 min and diluted. Insulin content was measured with the insulin ultrasensitive detection kit (Cisbio). Values are expressed as fold change over low glucose.

### Mouse IPGTT and ITT

All animal studies were approved by the Institutional Animal Care and Use Committee of UNC-Chapel Hill. Animal housing, care, and husbandry were overseen by the UNC Division of Comparative Medicine Animal Resources, which is accredited by the International Association for Assessment and Accreditation of Laboratory Animal Care. Prior to experiments, mice were multihoused in a temperature-controlled room with a 12:12 dark:light cycle and provided *ad libitum* access to water and standard chow.

To measure the glucose-lowering effect of GLP-1(7–36) peptide mice, IPGTT and ITT were performed on WT SvEv129/SJ and isogenic *Ramp3*^*−/−*^ male animals. *Ramp3*^*−/−*^ mice and genotyping have been previously described (15). For IPGTT, mice were fasted for 16 h and treated with saline or GLP-1 at 50 nmol/kg body weight (subQ) 15 min before glucose (2 g/kg body weight) intraperitoneal injection. Blood glucose was measured using a glucometer (TRUE MATRIX, Trividia Health, Inc) from the tail vein at baseline (before GLP-1 injection), 10, 20, 30, 60, and 120 min after glucose injection. Blood was collected at baseline and 15 min after glucose injection from the tail vein in EDTA cuvettes (Microvette CB 300, 16.444.100; Sarstedt). For ITT, mice were fasted for 5 h and treated with saline or GLP-1 at 50 nmol/kg body weight (subQ) 15 min before intraperitoneal injection of insulin (1 IU/kg). The blood glucose levels were measured from the tail vein at baseline (before GLP-1 injection) and 15 and 30 min after insulin injection using a glucometer.

### Detection of *in vivo* plasma insulin levels

During IPGTT, blood samples were collected in potassium EDTA cuvettes from the mouse tail vein at baseline and 15 min after glucose administration. Blood samples were centrifuged at 2000*g* for 10 min at 4 °C to separate the plasma (supernatant), which was collected in separate 1.5 ml Eppendorf tubes and stored at −80 °C. Using the mouse insulin ELISA kit (Ultrasensitive mouse insulin ELISA kit; Crystal Chem, 90080), plasma insulin levels were measured following the manufacturer’s instructions with two replicates for each sample.

### Data analysis

GraphPad Prism 10 (GraphPad Software, Inc) was used for analysis of all assays. cAMP, Ca^2+^ mobilization, β-arrestin recruitment, and internalization were fitted to a concentration–response curve using the three-parameter logistic equation. Potency (pEC_50_) and maximal (*E*_max_) values were obtained from this curve fit. Saturation-interaction curve data were fitted using a one-phase decay model, and binding data were fitted using either a model of association kinetics or the equation for a one-site binding model. LogRA values were calculated using

$$LogRA=\frac{{EC}_{50_{Ref}}E_{\max_{Test}}}{{E_{\max_{Ref}}EC}_{50_{Test}}}$$

Where Ref is the reference condition, and Test is that for which the LogRA is being calculated.

Error was propagated as previously described (46, 48).

Statistical differences were determined using a Student’s *t* test, one-way or two-way ANOVA, with Dunnett’s, Tukey’s, or Sidak’s *post hoc* test for multiple comparisons for parametric data, or the Kruskal–Wallis test with Dunn’s *post hoc* test for multiple comparisons for nonparametric datasets. A probability of *p* < 0.05 was considered significant. Values are reported as mean ± SEM of n repeats carried out in duplicate or quadruplicate.

## Data availability

All data are contained within the article.

## Supporting information

This article contains supporting information.

## Conflict of interest

The authors declare that they have no conflicts of interest with the contents of this article.
